# Risk determination and prevention of breast cancer

**DOI:** 10.1186/s13058-014-0446-2

**Published:** 2014-09-28

**Authors:** Anthony Howell, Annie S Anderson, Robert B Clarke, Stephen W Duffy, D Gareth Evans, Montserat Garcia-Closas, Andy J Gescher, Timothy J Key, John M Saxton, Michelle N Harvie

**Affiliations:** 1 Genesis Breast Cancer Prevention Centre, University Hospital of South Manchester, Southmoor Road, Wythenshawe, M29 9LT Manchester, UK; 2 The Christie, NHS Foundation Trust, Wilmslow Road, Manchester, M20 2QJ UK; 3 Breakthrough Breast Cancer Research Unit, Institute of Cancer Sciences, University of Manchester, Wilmslow Road, Manchester, M20 2QJ UK; 4 Centre for Public Health Nutrition Research, Division of Cancer Research, Level 7, University of Dundee, Ninewells Hospital & Medical School, Mailbox 7, George Pirie Way, Dundee, DD1 9SY UK; 5 Centre for Cancer Prevention, Wolfson Institute of Preventive Medicine, Queen Mary University of London, Charterhouse Square, London, EC1M 6BQ UK; 6 Manchester Centre for Genomic Medicine, The University of Manchester, Manchester Academic Health Science Centre, Central Manchester Foundation Trust, St. Mary’s Hospital, Oxford Road, Manchester, M13 9WL UK; 7 Division of Genetics and Epidemiology, Institute of Cancer Research, Cotswold Road, Sutton, SM2 5NG London, UK; 8 Department of Cancer Studies and Molecular Medicine, University of Leicester, University Road, Leicester, LE2 7LX UK; 9 Cancer Epidemiology Unit, Nuffield Department of Population Health, University of Oxford, Richard Doll Building, Roosevelt Drive, Oxford, OX3 7LF UK; 10 School of Health Sciences, Faculty of Medicine and Health Sciences, University of East Anglia, University Drive, Norwich, NR4 7TJ UK

## Abstract

Breast cancer is an increasing public health problem. Substantial advances have been made in the treatment of breast cancer, but the introduction of methods to predict women at elevated risk and prevent the disease has been less successful. Here, we summarize recent data on newer approaches to risk prediction, available approaches to prevention, how new approaches may be made, and the difficult problem of using what we already know to prevent breast cancer in populations. During 2012, the Breast Cancer Campaign facilitated a series of workshops, each covering a specialty area of breast cancer to identify gaps in our knowledge. The risk-and-prevention panel involved in this exercise was asked to expand and update its report and review recent relevant peer-reviewed literature. The enlarged position paper presented here highlights the key gaps in risk-and-prevention research that were identified, together with recommendations for action. The panel estimated from the relevant literature that potentially 50% of breast cancer could be prevented in the subgroup of women at high and moderate risk of breast cancer by using current chemoprevention (tamoxifen, raloxifene, exemestane, and anastrozole) and that, in all women, lifestyle measures, including weight control, exercise, and moderating alcohol intake, could reduce breast cancer risk by about 30%. Risk may be estimated by standard models potentially with the addition of, for example, mammographic density and appropriate single-nucleotide polymorphisms. This review expands on four areas: (a) the prediction of breast cancer risk, (b) the evidence for the effectiveness of preventive therapy and lifestyle approaches to prevention, (c) how understanding the biology of the breast may lead to new targets for prevention, and (d) a summary of published guidelines for preventive approaches and measures required for their implementation. We hope that efforts to fill these and other gaps will lead to considerable advances in our efforts to predict risk and prevent breast cancer over the next 10 years.

**Electronic supplementary material:**

The online version of this article (doi:10.1186/s13058-014-0446-2) contains supplementary material, which is available to authorized users.

## Introduction

Breast cancer remains a major public health problem. The incidence is rising in most countries and is projected to rise further over the next 20 years despite current efforts to prevent the disease [1]-[4]. The increased incidence is not surprising since there has been, in most countries, an increase in numbers of women with major breast cancer risk factors, including lower age of menarche, late age of first pregnancy, fewer pregnancies, shorter or no periods of breastfeeding, and a later menopause. Other risk factors which add to the burden of breast cancer are the increase in obesity, alcohol consumption, inactivity, and hormone replacement therapy (HRT) [4]. The impact of hereditary breast cancer has also increased. For example, it is estimated that the penetrance of the breast cancer 2 (*BRCA2*) founder mutation in Iceland increased fourfold over the last century, and the cumulative incidence of sporadic breast cancer by age 70 also increased fourfold, from 2.5% to 11% of the population, over the same period [5]. Birth cohort effects have also been seen for both *BRCA1* and *BRCA2* in other countries [6],[7]. These data suggest that both familial and non-familial risks have increased. The Collaborative Group on Hormonal Factors in Breast Cancer (2002) estimated that the cumulative incidence of breast cancer in developed countries would be reduced by more than half, from 6.3 to 2.7 per 100 women, by age 70 if women had on average more children and breastfed for longer periods as seen in some developing countries [8]. Given global increases in population growth and the strong evidence that a woman’s ability to control her fertility may improve her social, economic, and overall health, it is not considered desirable to increase the birth rate per woman or to encourage pregnancies at a very young age. However, breastfeeding can and should be encouraged for many reasons, including possibly for the reduction of breast cancer risk. Many of the risks of reproductive factors are related to the effects of estrogen as demonstrated by the reduction in breast cancer incidence after an early oophorectomy, by inhibition of the estrogen receptor (ER) by using selective estrogen receptor modulators (SERMs) such as a tamoxifen or raloxifene [9], or by blocking estrogen synthesis by using aromatase inhibitors (AIs) such as exemestane [10] and anastrozole [11],[12].

A paradigm for preventative therapy (chemoprevention) is cardiovascular disease (CVD). The introduction of drugs that suppress cholesterol synthesis, modify platelet aggregation, or lower blood pressure has led to a steady decline in CVD over the past three decades, such that deaths from CVD in women less than 85 years old fell below those for cancer in 1999 [13]. The cardiovascular community is helped by the reduction of a major risk factor (smoking) and having easy-to-measure, repeatable biomarkers (cholesterol and blood pressure). CVD deaths are also reduced by optimal treatment of disease once it arises; this is also true for breast cancer treatment, in which (as a result of the introduction of screening and optimizing treatments) deaths have decreased by approximately one third over the past 20 years. This is a major advance for breast cancer; however, primary prevention has not occurred at the population level in contradistinction to CVD.

The fraction of breast cancer cases attributable to lifestyle and environmental factors in the UK was estimated to be 26.8% in 2010 [14], and a recent review suggests that half of breast cancer cases may be prevented if chemoprevention is applied in appropriate at-risk populations and the major modifiable risk factors, including achieving and maintaining a healthy weight, regular physical activity (PA), and minimal alcohol intake, are instituted [4]. Thus, there are further possibilities of important reductions in breast cancer incidence. However, major gaps exist in our knowledge to determine the risk of breast cancer accurately in order to apply these approaches to appropriate populations of women.

This review is an expansion and update of a brief review published in the Gap Analysis in 2013 of breast cancer research overall [1]. Besides summarizing new data published over the past year, this review has enabled us to give more comprehensive summaries of risk factors, approaches to prevention, and how understanding the biology of the breast may lead to new approaches to risk and prevention and also to expand on the all-important area of how to implement current risk prediction and preventive measures in the population (Table 1).

**Table 1:** **Major gaps in our knowledge concerning risk assessment and prevention of breast cancer**

A. Gaps in risk estimation
A1. The best standard model to estimate risk in the general population and in women at high risk
A2. What additional factors will give maximal improvement in a model?
A3. Prediction of risk in the proportion of women with none of the current risk factors
B. Gaps in preventive therapy and lifestyle prevention
B1. Prediction of women who will benefit from current preventive therapy
B2. New agents for women who will not benefit from current preventive therapy
B3. Optimal measures for weight control and exercise: timings of this in the life course, who to target, and type of interventions
C. Gaps in understanding the biology of breast cancer risk
C1. Mechanisms of the effects of pregnancy on risk
C2. Mechanism of the lack of involution in some breasts with menopause?
C3. Mechanism of energy restriction on reduction of risk
D. Gaps in implementing known preventive measures
D1. Determination of the approximately 10% of women at high and moderate risk in populations
D2. How to make preventive therapy available to the subset of women who will benefit
D3. Optimal weight control and exercise programs for women at any age and in all countries and how we engage individuals in cancer prevention throughout the life course

## Methods of risk assessment

Models and scoring systems have been developed either to predict the probability that a person carries a mutation in the *BRCA1/2* genes, which is relevant to relatively small numbers of women with strong family histories, or to predict breast cancer risk over time [15],[16]. Computer models such as BOADICEA (The Breast and Ovarian Analysis of Disease Incidence and Carrier Estimation Algorithm) and BRCAPRO (risk estimator for breast and ovarian cancer) [17] and scoring systems perform well for predicting *BRCA1/2* mutation carrier probability, which is important in deciding whether to perform a genetic test [18],[19].

Of relevance to all women, several models have been developed to predict risk of breast cancer over time (for example, 5-year, 10-year, or lifetime risks). These predict the probability that a woman in the population with a particular combination of risk factors will develop breast cancer [14]-[16]. The tested models include the Tyrer-Cuzick [20] and Gail [21] models, both of which include family history and non-familial risk factors, BOADICEA [22], a modification of the Claus model to include non-familial risk factors [23], the Rosner-Colditz model [24], and several others, many of which require further validation [16].

The Gail model includes these risk factors: age at menarche, age at first live birth, number of previous breast biopsies, benign breast disease, and number of first-degree relatives with breast cancer. Studies indicate that the Gail model is well calibrated in regularly screened American women [25] and when using updated breast cancer incidence [26]. However, recent studies in the UK and US suggest that it may under-predict actual risk relative to the Tyrer-Cuzick model [27]-[29], possibly because of the limited family history and not including age of onset of cancer in the family whereas the Tyrer-Cuzick model also includes second-degree family history, age of onset of cancer, and use of HRT.

Although current models can give an accurate estimation of lifetime risk (for example, we can tell a woman, with some accuracy, that she has a 1 in 3 lifetime risk of breast cancer), we cannot tell her whether she is the one who will develop the disease or whether she is one of the two women who will not. To fill this gap in our knowledge, there is great interest in adding other risk factors to current models, such as mammographic density [30],[31], single-nucleotide polymorphisms (SNPs) [32],[33], estimation of hormone levels [34], and lifestyle factors in order to test whether they improve the accuracy of risk prediction in the female population. Here, we examine recent progress made in improving available breast cancer risk prediction models.

### Improving risk estimation - mammographic density

**Figure 1:**
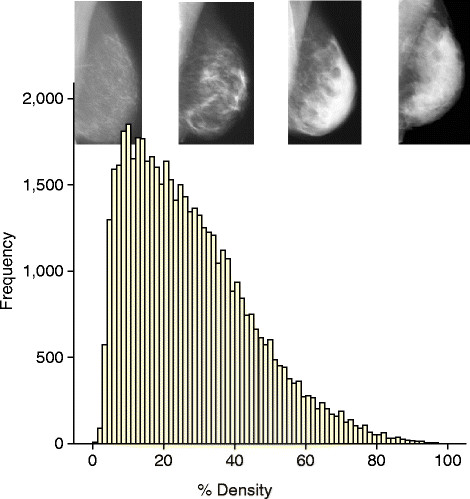
**An example of the distribution of visually assessed percentage density of the breast.** The sample consists of 50,831 women between 46 and 73 years of age. Density was estimated in two views of each breast on a visual analogue scale, and the four readings were combined to give a single value per woman [54].

The available data on mammographic density in relation to breast cancer risk have been reviewed recently [30],[31]. Dense tissue on the mammogram is white, whereas fat tissue is radio-lucent and appears black. An overview of 42 studies of visually assessed mammographic density (the proportion of the breast as a percentage which appears white) indicated that the relative risk of breast cancer for women with 70% or more density was 4.64-fold greater compared with women with less than 5% density [35]. In this report, the magnitude of the risk was greater using percentage density than for other visual methods of density estimation, such as Wolffe patterns or the Breast Imaging Reporting and Data System (BI-RADS) classification, which divides density into four visually assessed categories and is widely used in the US. The distribution of visually assessed mammographic density is shown in Figure 1.

Four studies have already assessed whether adding a measure of mammographic density improves risk estimation compared with the estimation using standard models alone. A standard measure of improvement of risk assessment is the C-statistic. This is the area under the receiver operating curve (AUC), which in turn is a reflection of the sensitivity and specificity of the model. The higher the C-statistic (AUC), the greater the discriminatory accuracy of the model. An AUC of 0.5 identifies a model whose discriminatory accuracy is no better than chance alone, whereas an AUC of 1.0 identifies a model with perfect discriminatory accuracy. In practice, AUCs of 0.7 or 0.8 are consistent with good discriminatory accuracy [15].

Tice and colleagues [36] estimated adding the BI-RADS assessed density to the Gail model. The C-statistic for the Gail model in this study was 0.67, but adding density to the model modestly increased the C-statistic to 0.68, although this small increase in discriminatory accuracy was significant (*P* <0.01). Barlow and colleagues [37] reported an increase of the C-statistic from 0.605 (95% confidence interval (CI) 0.60 to 0.61) to 0.62 (95% C1 0.62 to 0.63) also by adding BI-RADS density to the Gail model. Chen and colleagues [38] demonstrated that adding percentage density to the Gail Model 2 significantly (*P* = 0.015) increased the C-statistic, from 0.602 to 0.664. Tice and colleagues [39] performed a second study of adding BI-RADS to a modification of the Gail model and reported a C-statistic rise from 0.61 to 0.66. These studies are important in that there was an improvement, albeit modest, in discriminatory accuracy in all of them.

It should be borne in mind that owing to the correlations among breast cancer risk factors, the addition of a new risk factor, however powerful, to a model already containing several risk factors will invariably make a modest difference to prediction measures such as AUC. Whereas some studies have suggested that density adds little to risk prediction [40], some find AUCs for density or another breast composition measure alone of 0.6 to 0.8 [41]-[44], which is similar to those observed for the Gail and other models.

Although the improvement in the C-statistic shown in these studies is modest, a more relevant measure of the utility of adding density information to risk models is how much it improves the ability to identify women at different levels of absolute risk for breast cancer (for example, re-classification of women crossing threshold risk levels set for public health interventions such as enhanced screening or chemoprevention). Further validation of risk models, including BI-RADS or other density measures such as volumetric approaches in prospective cohort studies, is needed to assess potential value of density in risk-stratified prevention or screening programs.

One method of density estimation, the interactive thresholding technique known as CUMULUS developed in Toronto [45], determines the area of dense and non-dense tissue, unlike visual techniques outlined above, and is widely regarded as a gold standard method for estimation of density. A meta-analysis of 13 case-control studies using this technique indicated that the association of density with risk was strong. Perhaps surprisingly, the risk prediction was better for dense area as a percentage of the whole breast rather than absolute dense area [46]. There remains a need to assess whether some measure of CUMULUS density adds to the predictive accuracy of standard models. CUMULUS is time-consuming and requires specialized training, and the technique will require greater automation to be useful on a population basis (Nickson and colleagues [47]).

Methods are being developed to assess the volume of dense and non-dense tissue in the breast and may be more relevant not only because density is a volume but because they can be partially or fully automated with the potential for use in populations of women. The first reported estimation of the relationship of volumetric density to standard risk factors was by Shepherd and colleagues [48], who used a technique called single x-ray absorptiometry. In their study, the C-statistic for risk factors alone was 0.609, which significantly increased to 0.667 when log fibro-glandular volume was added to standard risk factors. The study was performed by using analogue mammograms. Newer automatic techniques - such as Quantra (Hologic, Inc., Bedford, MA, USA) and Volpara (Matakina International, Wellington, New Zealand) - are designed for use with modern digital mammograms and are fully automatic. How they add to standard models is being tested, but studies already demonstrate that they are consistent with magnetic resonance imaging measures of volumetric density [49],[50].

### Improving risk estimation - single-nucleotide polymorphisms

Mutations in high-risk breast cancer genes such as *BRCA1/2* affect only small numbers of women, whereas variation in lower-impact, common susceptibly loci or SNPs can be responsible for a larger percentage of cancers in the population. Although it has been predicted for some time that risk would be related to polygenic inheritance of common low-penetrance loci [51], these have only recently been identified. SNPs are, by definition, common alterations in the DNA code that are mostly thought to be non-functional variants that frequently occur outside functional genes. Relative risks from SNPs are small (maximum risk is around 1.43-fold) and many have effects of less than 1.1-fold. Recent reports of ‘risk’ SNPs are a result of large-scale multinational collaborations involving tens of thousands of breast cancer cases and appropriate controls. Such large-scale studies are required since each SNP is associated with a small increase or decrease in risk. However, in combination (for example, through polygenic risk scores based on the average of the number of risk alleles weighted by the relative risk associated with each allele), combined SNPs can be associated with substantial increases or decreases in risk. The number of validated SNPs associated with breast cancer risk is currently over 70, but it is thought that there may be hundreds more that affect breast cancer risk [32].

Based on the first few SNPs identified, studies were performed to determine how they might add to the Gail model. All studies showed some improvement in the C-statistic when SNP scores and the Gail model were combined. Mealiffe and colleagues [52] using seven SNPs reported an increase in AUC from 0.58 to 0.61 (*P* = 0.001), Wacholder and colleagues [53] using 10 SNPs reported an increase in the AUC from 0.58 to 0.62 (*P* <0.001), and Gail [54] predicted an increase in the C-statistic from 0.61 to 0.63. More recently, Dite and colleagues [55] included seven SNPs and reported an increase in AUC from 0.58 to 0.61 (*P* <0.001).

**Figure 2:**
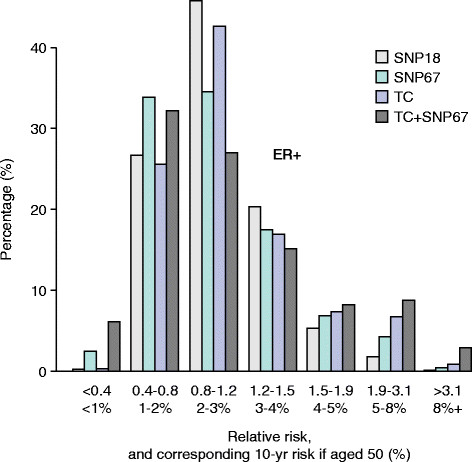
**Estimation of the effect on the distribution of Tyrer-Cuzick scores by adding the results of 18 or 67 single-nucleotide polymorphisms (SNPs) in 10,000 women [**[53]**].** Adding SNPs increases the number of women in high- and low-risk groups. ER, estrogen receptor; SNP 18 and SNP 67, distribution using SNPs alone; TC, the Tyrer-Cuzick score alone; TC + SNP67, distribution of the combined score.

An additional way to determine the value of adding SNPs to risk models is to assess changes in risk group stratification before and after adding SNPs. For instance, increasing the numbers of women estimated to be truly at high or low risk would be of value clinically. All the studies outlined above resulted in changes in classification to higher and lower risk categories resulting in a ‘widening’ of the risk distribution curves. For example, in the study by Comen and colleagues [56], a combination of 10 risk SNPs and the Gail model resulted in 20% of women being re-classified into a lower and 20% into a higher risk group as defined by quintiles. More recently, Brentnall and colleagues [57] and Evans and colleagues [58] estimated the effect on risk of combining 18 or 67 SNPs and the Tyrer-Cuzick model (Figure 2). Adding more SNPs changed the risk distribution so that more women were in the high- and low-risk groups, respectively (Figure 2).

The studies outlined above highlight the prospects of using SNPs for improved risk prediction in high-risk clinics and in the general population. Further improvements may come from introducing more SNPs and the prospects of being able to predict the risk of specific breast cancer subtypes, such as ER^+^[59], ER^−^[60], grade III [61], and triple-negative [62] tumors, separately, knowledge of which could direct preventative approaches [63].

### Improving risk estimation - hormone measurements

Large studies with long-term follow-up indicate that many hormones and growth factors are associated with an increased risk of breast cancer. The important question is whether any of them could be incorporated into models of breast cancer risk prediction. The Endogenous Hormones and Breast Cancer Collaborative Group reported that risk of breast cancer was related to steroid hormones such as estradiol, testosterone, and sex hormone-binding globulin in pre- and post-menopausal women and was recently confirmed in the European Prospective Investigation into Cancer study [64]-[67]. The relation of body mass index (BMI) with risk is attenuated by adjusting for estrogen, but the relation of estrogen with risk is not attenuated by adjusting for BMI. This is what would be expected if estrogen mediates the effect of BMI [64]. Thus, estrogens may explain the increased risk of breast cancer in obese post-menopausal women, although this does not preclude other hormones and cytokines from mediating the effects of estrogen (which may be more readily measurable) or other mechanisms by which overweight and obesity might affect risk [64],[68].

The use of hormone measurements in breast cancer to incorporate into risk models is attractive. However, measurement, particularly in post-menopausal women, is problematic because of assay variation related to low hormone levels and other unknown causes of variation in hormone levels over time [69]. Nevertheless, Jones and colleagues [70] demonstrated that change in estradiol and testosterone may be good biomarkers of the effectiveness of weight loss and this is supported by recent data from the Nurses’ Health Study [71]. Other growth factors/hormones such as insulin-like growth factor-1 (IGF-1) and prolactin are associated with breast cancer risk, particularly in post-menopausal women, and may possibly be useful in models, although the risk increases between high and lower risk groups of hormone concentrations are relatively small [72]-[75].

### Improving risk estimation - other methods

New biomarkers for risk prediction are likely to come from measures in blood or tissues by a variety of techniques. At present, it appears that none of these is ready for incorporation into the standard models, but given the pace of advance they are likely to be in the near future. Examples of some current approaches include the development of assays for serum antibodies against epithelial antigens [76], gene expression in peripheral blood white cells [77], blood epigenetic markers [78], and developments in high-throughput proteomics [79] and adductomics [80]. Incorporating new risk markers into risk models may not be straightforward since extensive validation will be required and potential interactions with known existing factors will need to be carefully evaluated.

## Breast cancer prevention

What can we advise women to do with respect to prevention? Recent reviews focus on various aspects of prevention, including SERMs and AIs for the chemoprevention of ER^+^ cancers [81],[82], chemoprevention for ER^–^ cancers [83],[84], and lifestyle changes [4],[85],[86]. These reviews are helpful in pointing out some areas that are potentially clinically useful and others where far more investigational work is required.

There is probably sufficient evidence from the randomized trials for the use of SERMs and AIs for use in women at high and moderate breast cancer risk [9],[11] and sufficient observational data to advise weight control, exercise, and moderation of alcohol intake [4],[86]. In this section, we review the data which support these suppositions for each of the approaches to prevention; in the next section, we review possible new investigational avenues.

### Preventative therapy (chemoprevention)

There have been nine randomized trials of SERMs [9] and two trials of AIs [10],[11] mainly in women at increased risk of breast cancer but also in women with osteoporosis or heart disease (raloxifene). In the SERM trials, 83,399 participants were included with 306,617 years of follow-up over an average period of 65 months. The overall reduction in all breast cancer (including ductal carcinoma *in situ*) using tamoxifen 20 mg per day was 38% (*P* <0.0001) [9] with an estimated 10-year reduction in cumulative incidence from 6.3% in the control group to 4.2% in the SERM groups. This overview included the SERMs lasofoxifene and arzoxifene, which are not undergoing further development by their respective drug companies. This leaves tamoxifen and raloxifene as the two SERMs in clinical practice. These were compared in a randomized trial (the Study of Tamoxifen and Raloxifene, or STAR, trial) [87]. Tamoxifen was significantly superior to raloxifene in longer-term follow-up for preventing invasive breast cancer (relative risk raloxifene/tamoxifen 1.24, 95% CI 1.05 to 1.47). Nonetheless, raloxifene was associated with fewer side effects than tamoxifen, particularly with respect to the uterus, and may be preferable in post-menopausal women.

When given after surgery to prevent relapse of breast cancer, AIs are generally superior to tamoxifen. This led to the initiation of two placebo-controlled trials in post-menopausal women at increased breast cancer risk. One tested the AI exemestane and reported a reduction of breast cancer risk of 65% after 5 years of treatment [10]. In the other trial (International Breast Cancer Intervention Study II, or IBIS II), anastrozole was compared with placebo [11]. In that study, 3,864 post-menopausal women between 40 and 70 years of age at increased risk of breast cancer were randomly assigned to anastrozole 1 mg per day or placebo for 5 years. A recent report indicates that the incidence of breast cancer was reduced by 53% (hazard ratio 0.47, 95% CI 0.32 to 0.68) by use of anastrozole. Compared with SERMs, AIs are not associated with an increased risk of thromboembolic disease and uterine problems, including cancer, but are associated with increased mild to moderate bone/muscle pain and reduced bone density.

Additional hormonal approaches to prevention surround the use of HRT. Results from the Women’s Health Initiative (WHI) randomized controlled trial of premarin and medroxyprogesterone acetate indicate that the combination given after menopause increases breast cancer risk [88], a result supported by many observational studies. After the publication of the WHI study, many women stopped HRT and it has been suggested by some to have been associated with a reduction in the incidence of breast cancer, CVD, and venous thrombosis as well as potential considerable savings in health resources [89]. However, the magnitude of these associations, as well as the question of whether a cause-and-effect relationship exists, remains controversial. In contrast, estrogen-only HRT using premarin resulted in a reduction of the incidence and deaths from breast cancer in the second WHI trial performed in women with a previous hysterectomy [90]. This result is supported by some, but not all, observational studies and indicates that premarin may be regarded as a breast cancer preventive agent [91].

### Lifestyle

The World Cancer Research Fund/American Institute for Cancer Research (WCRF/AICR) has estimated that over 40% of post-menopausal breast cancer could be prevented by reductions in alcohol, excess body weight, and inactivity [92]. These estimates differ from those suggested by others as outlined above [4],[14], but all of the estimates point in the same direction and indicate the importance of lifestyle throughout the lifespan and the challenge of finding ways to support women to achieve healthy ways of life.

### Energy restriction/weight control

Strong observational data indicate that weight gain in the premenopausal period and being overweight or obese after menopause increase breast cancer risk [4],[93]. In a meta-analysis, Renehan and colleagues [93] estimated that for each 5 kg/m^2^ increase in BMI the risk of breast cancer was increased by 12%. Evidence from two large observational studies indicates that pre- or post-menopausal weight loss reduces the risk of post-menopausal breast cancer. In the Iowa Women’s Health Study, sustained weight reduction of 5% of body weight reduced post-menopausal breast cancer risk by 25% to 40% compared with women who continued to gain weight [94]. In the Nurses’ Health Study, post-menopausal women who did not take HRT and maintained a body weight reduction of 10 kg or more had a 50% reduction in the risk of breast cancer [95]. There is some evidence from the National Surgical Adjuvant Breast Project P-I and STAR SERM trials that weight reduction after the age of 35 is also effective [96]. It is important to emphasize the other well-known beneficial effects of weight control, including the reduction of diabetes [97],[98] and CVD [99],[100]. Modest weight loss of 5% to 10% will reduce the risk of diabetes by up to 60% and can reduce low-density lipoprotein cholesterol by 15% and triglycerides by 20% to 30%, increase high-density lipoprotein cholesterol by 8% to 10%, and reduce blood pressure by around 5%. These changes in CVD risk markers suggest a 30% or greater reduction in risk of CVD.

### Dietary components and prevention

There is great interest in determining whether components of diets such as saturated fat content or the amount of fruit and vegetables is related to the risk of breast cancer. A randomized trial performed by the WHI of reduction of the proportion of fat in the diet resulted in a non-significant 8% reduction in the risk of breast cancer, but there was some confounding with weight loss [101]. After surgery for breast cancer, where dietary interventions were performed in addition to standard adjuvant therapy, reduction of fat was associated with a 23% reduction in recurrence. This study was also confounded by weight loss in the intervention arm and thus in both studies the reason for the effects on risks is not clear [102]. There was no advantage to an increase of fruit and vegetable intake in another large randomized adjuvant trial [103]. Recent large pooled analyses have suggested that both dietary intake of vegetables and circulating concentrations of some carotenoids may be inversely associated with the risk for ER^–^ breast cancer but not with the risk for ER^+^ disease. This topic requires further investigation [104],[105]. Whereas intervention studies give little support for the preventive efficacy of specific dietary components, prospective cohort studies provide indications that adherence to dietary guidelines and certain types of diet may impact on breast cancer risk. Adherence to dietary and lifestyle guidelines appears to be beneficial. In a study from Canada [106], adherence to the American Cancer Society (ACS) and WCRF/AICR dietary/lifestyle guidelines appeared to be beneficial: 49,613 women completed dietary and lifestyle questionnaires, and adherence was associated with a 31% reduction of breast cancer estimated over 16 years compared with women who did not follow the guidelines. The guidelines include advice on weight control, PA, alcohol intake, and intake of red meat, vegetables, fruit, and sodium. In another study, the WHI reported the effects of adherence to ACS guidelines in 65,838 post-menopausal women and indicated that adherence to guidelines reduced breast cancer risk by 22% after 12.6 years of follow-up [107].

Adherence to dietary types may also affect risk. For example, in the California Teachers Study, data from 91,779 women were analyzed according to predominant dietary pattern by using principal component factor analysis [108]. A greater consumption of plant-based foods was associated with a 15% reduction in breast cancer risk (85% CI 0.76 to 0.95). A systematic review of dietary patterns and breast cancer was performed by Albuquerque and colleagues [109], who concluded that a Mediterranean dietary pattern and diets composed largely of vegetables, fruit, fish, and soy are associated with a decreased risk of breast cancer. Risk reduction may also be helped by appropriate intakes of dietary fiber, fruit, and vegetables [110]-[114].

### Physical activity

More than half of the US population does not meet the recommended PA guidelines. In addition, the most recent Health Survey for England [115] showed that over 40% of adult women (at least 19 years old) are not meeting current guidelines of 150 minutes of moderate or 75 minutes of vigorous PA per week [116]. The WCRF/AICR Expert Report [117] described the evidence for an inverse association between PA and breast cancer risk as ‘probable’ and ‘limited - suggestive’ for post- and pre-menopausal women, respectively. A more recent review of 73 observational studies indicated that moderate to vigorous PA reduces breast cancer risk by an average of 25% in pre- and post-menopausal women compared with inactive women [118]. The strongest inverse associations with breast cancer risk were observed for recreational PA, lifetime PA, post-menopausal PA, and participation in moderate to vigorous PA. There was also evidence of dose-response relationships, with higher volumes of PA associated with greater risk reduction, but with the most pronounced reductions in risk being observed in lean versus obese women. The optimal level of PA for breast cancer risk reduction is unclear, however, and may be greater than current recommendations [118]. A major limitation of observational studies is the heterogeneity of self-report questionnaires that have been used to measure PA. The use of more objective measures, such as 7-day accelerometry, would provide more robust PA data. There is a clear need for randomized controlled trials which include clinical end-points or biomarkers on the causal pathway, but designing such trials is challenging because of the large sample size required and the expense of collecting long-term follow-up data.

### Alcohol

It is estimated that breast cancer risk is increased by 7% to 10% for each one-unit increase in intake of alcohol per day (a unit is half a pint of 4% strength beer or cider or 25 mL of 40% strength spirits, and a small 125-mL glass of 12% strength wine is 1.5 units). In the Nurses’ Health Study, women who consumed 4 to 9 units per week were 15% more likely to develop breast cancer compared with never drinkers [119]. Women with the highest alcohol intake (of at least 27 units per week) were 51% more likely to develop breast cancer compared with non-drinkers. These studies suggest that women who want to minimize their breast cancer risk should not be drinking more than one unit daily and probably have at least two alcohol-free days weekly. Studies show that the negative effect of alcohol may be abrogated by adequate dietary folate intake (rather than supplements) and should be pointed out as a preventive measure for women who find reduction in alcohol intake difficult [120]. Better life expectancy associated with moderate alcohol intake compared with none in a large meta-analysis should be balanced against recommending zero intake [121].

It is important to be aware that lifestyle prevention includes not only middle- and late-age women but younger women after menarche. Animal experiments and modeling of the reproductive events in women indicate that the most susceptible period for carcinogenesis is during the period between menarche and first pregnancy [122],[123]. In women, this susceptibility is highlighted by the increase in premalignant lesions in the breast of women who drank alcohol or smoked (or both) during this period of early life [124].

## The biology of risk and prevention as an indicator of potential new approaches

**Figure 3:**
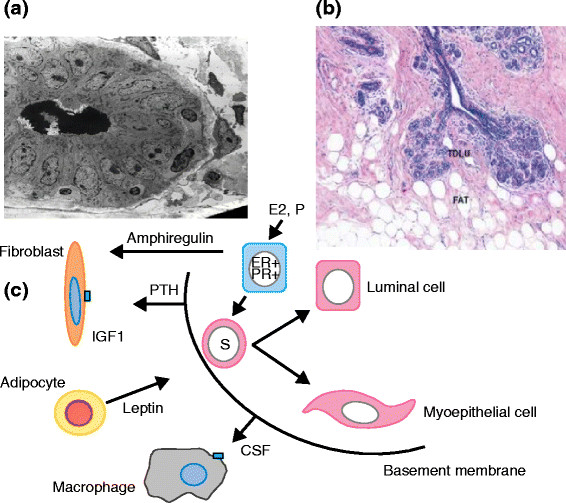
**Features of the normal breast. (a)** Electron micrograph of a ductule of the breast. **(b)** Section of lobules of the breast showing a relationship with collagenous and fatty stroma. Reprinted with permission from the American Association for Cancer Research [166]. **(c)** A simplified cartoon of reported potential interactions between three cell types in the stroma and the epithelium of the breast. CSF, colony-stimulating factor; ER, estrogen receptor; IGF1, insulin-like growth factor 1; PR, progesterone receptor; PTH, parathyroid hormone; TDLU, terminal duct lobular unit.

One way to develop new approaches to prevention is to assume that understanding the biological basis of breast development will give indications of potential targets for therapeutic interventions. Great insights into the mechanisms of breast development *in utero* and at puberty, particularly in the rodent mammary gland, have been discovered and are summarized in recent reviews [125],[126]. They highlight the crucial importance of epithelial-stromal interactions for normal breast development and of the individual cell types within the stroma, including immune cells, fibroblasts, or adipocytes. Importantly, it has been shown that experimental inhibition of any one of these interactions results in lack of breast development and this has implications for our thinking about approaches to prevention (Figure 3).

The experiments outlined above cannot be performed in humans. However, another approach to the development of prevention is understanding the biological mechanisms of risk factors for breast cancer. Here, we discuss some examples which support this view with respect to estrogen and the breast, early and late first pregnancy, menopausal involution of epithelial cells, mammographic density, and mechanism of the effects of energy restriction and exercise.

### Estrogen and the breast

The most successful preventative approach to breast cancer to date, reducing the effects of estrogen on the breast, has come from an understanding of the biology of the ER and the knowledge that estrogen is synthesized in the breast and elsewhere after ovarian function decreases at menopause. These data have led to the introduction of the SERMs (tamoxifen and raloxifene) and the potential introduction of AIs (exemestane and anastrozole) for breast cancer prevention. Tamoxifen acts by blocking the ER but under certain circumstances can change to being a partial agonist via the ER and this may limit its preventive utility since in some women at increased risk it appears to increase mammographic density [127]. The development of orally active ER downregulators similar to fulvestrant (which has to be given intramuscularly, thus limiting its preventive utility) may be superior to tamoxifen (for example, ARN-810, NCTO1823835) [128]. Another potential way to enhance the therapeutic ratio of tamoxifen is to use low doses or to combine tamoxifen with retinoids such as fenretinide; studies of these approaches are under way in prevention trials in Italy [129]. Another approach may be a combination with low-dose aspirin, which has some minor preventive effects on breast cancer risk but would help combat the increased risks of thromboembolic disease with tamoxifen.

### Mimicking the protective effects of an early first pregnancy

Recent insights into the effects of early first pregnancy of the normal breast in young women give clues to how we might mimic this effect therapeutically. Since the demonstration that ER^+^ and progesterone receptor-positive (PR^+^) cells in the normal breast rarely proliferate [130], it has been shown, for example, that progesterone binds to its receptor on the PR of the epithelial cell and stimulates the synthesis and release of paracrine mediators such as Rank (receptor activator of nuclear factor-kappa-B), Wnt (wingless related integration site), and growth hormone, which in turn stimulate adjacent stem and progenitor cell expansion [131],[132]. Recently, it was shown that early first pregnancy in women reduces the number of PR^+^ cells and downregulation of paracrine mediators, resulting in a reduction of the stem/progenitor cell compartment [133]. These data suggest that modulating the effect of progesterone by the use of antiprogestins should be explored for breast cancer prevention [134].

### Establishing the cause of the inverse association between childhood/adolescent obesity and lower risk of breast cancer

Observational data have linked diet and growth in height in childhood and dietary exposures during early adulthood (that is, between menarche and first full-term pregnancy to later risk of breast cancer). These studies have either used retrospective recall of early life exposures from adults or prospectively assessed short-term effects on surrogate risk markers like benign breast disease [135]. Studying lifestyle exposures in this period is a challenge which has understandably received less research attention than exposures later in life. The period between menarche and first full-term pregnancy is a priority for research since risk can accumulate rapidly in this period until terminal differentiation that accompanies first pregnancy.

Key observations which deserve further study are the reduced breast cancer risk with a higher BMI in early adulthood (that is, at the age of 18 to 21), reported from numerous prospective studies among Caucasian [136],[137], black [138], and Asian [139] populations. This observation is partly explained by smaller adult weight gains, which are consistently reported among heavier young women [140]-[143]. Other possible mechanisms which may put heavier women at lower risk than their lean counterparts include higher estrogen levels, which may upregulate the *BRCA1* tumor-suppressor gene, earlier differentiation of breast tissue [9], subsequent lower IGF-1 levels in adulthood [144], and a slower pubertal growth and sexual maturation despite their early menarche [135]. Increased irregular cycles are often cited as a likely protective mechanism but are not supported by available data [145]. Likewise, height velocity has been linked to risk of breast cancer [146] and benign breast disease [147], which in turn may be linked to dietary patterns which are high in animal versus vegetable protein and lower in fiber and isoflavones [148].

### Reversing the promotional effects of late pregnancy

Late pregnancy is a major driver of the worldwide increase in breast cancer incidence. Over half of women in the UK have their first pregnancy over the age of 30, and thus understanding the mechanism of its effect on risk is of great importance. It seems likely that the breasts of older fertile women harbor early pre-cancerous lesions. One mechanism in which these may be stimulated is as a result of immunological processes that occur during post-partum breast involution. Lyons and colleagues [149] demonstrated an increase in cyclooxygenase 2 during involutional macrophage infiltration and showed that ibuprofen reduces post-partum breast cancer in these models. Ibuprofen might be tested in women at high risk because of late pregnancy and a positive family history [148],[149]. Premalignant lesions in the breast have indeed been detected by review of serial sections of the breasts at post-mortem of older premenopausal women and found to be present in up to one third of women [150],[151]. It is clear that most do not progress to breast cancer since the incidence of the disease is not that high. Recently, Haricharan and colleagues [152] demonstrated that the signal transduction molecule pSTAT5 (phospho-signal transducer and activator of transcription 5) is activated by inhibiting apoptosis in premalignant lesions that progress to forming cancer. Inhibitors of this pathway are in the clinic and ultimately could be used for prevention [153].

#### Failure of menopausal breast involution

The lobules of the breast undergo involution after menopause. However, Wellings and colleagues [154] reported atypical premalignant lobules which persisted after menopause where menopausal regression might be expected. Investigators at the Mayo Clinic noted, by careful histological examination of biopsies of the breast of post-menopausal women, that the breast lobules in some women did not undergo post-menopausal involution and that these women were at high risk of subsequent breast cancer [155]. As a measure of the importance of this observation, the authors investigated how the lack of involution compared with risk prediction of the Gail model in this group of women. The C-statistic for the Gail model of the patients studied was 0.60. For lobular involution (or not), the C-statistic was 0.66. Combining Gail risk and involution did not change the latter figure [156]. There are, as far as we are aware, no published data on the mechanism of lack of post-menopausal involution but this may be similar to the lack of involution after a pregnancy [152]. The reduction of apoptosis reported in animal models of pregnancy involution was reported in women [157]. In the clinic, there are agents to enhance apoptosis, such as ABT-263, with potential for transfer to prevention if toxicity could be reduced [158].

#### Mechanism of mammographic density

Some studies show that the rate of the well-known decline of mammographic density with age is slower in some women and indicates higher breast cancer risk [159],[160]. Methods to reduce density may prevent breast cancer. As proof of principle of this hypothesis, Cuzick and colleagues [127] demonstrated in the IBIS-I prevention trial that women who had a more than 10% reduction in density with tamoxifen had a 70% reduction in risk of breast cancer risk but that for women with less or no reduction in density there was no reduction in risk. Investigation of the reasons for the lack of effect of age and of tamoxifen on some breasts is clearly important [161].

Gene expression profiles of fibroblasts derived from dense and non-dense areas of the breast indicate marked differences in expression. Expression of genes associated with inflammation (such as c-Jun N-terminal kinases, or JNK) and several signaling pathways is upregulated and suggests the use of, for example, JNK inhibitors, already in the clinic for treatment of overt disease [162],[163]. Some fibroblasts in dense areas resemble cancer-associated fibroblasts in their signaling pathways and production of extracellular aligned collagen, all potential targets for prevention [164].

#### Energy restriction mimetics

Energy restriction is well known to increase longevity in several types of organisms, in part by reducing the incidence of cancer. It acts predominantly by reversing the effects of obesity on inflammation, certain signal transduction pathways, and insulin/IGF-1 [165]. Obesity is associated with macrophage infiltration and activation in fat, which in turn results in cytokine production and increased aromatase activity and estrogen production [166],[167]. Obesity also results in reduced insulin sensitivity and altered signal transduction pathways, such as P13Kinase and mammalian target of rapamycin (mTOR), and in mitochondrial metabolism [168],[169]. Some agents which beneficially reduce activity of these pathways such as mTOR inhibitors are already in the clinic, and others such as metformin and SIRT 1 activators such as resveratol and other activators of sirtuins are under investigation [170]. Doubt has been cast on the value of metformin [171], giving added importance to the randomized trial of adjuvant metformin instigated by Goodwin and colleagues [172].

### Physical activity

Several biological mechanisms have been proposed to explain the inverse association between PA and breast cancer risk. Although regular exercise may delay the onset of menarche, increase the length of the menstrual cycle, or increase the number of anovulatory cycles, hence reducing exposure to sex hormones, prospective intervention studies suggest that high levels of exercise may be needed to induce menstrual cycle changes [173],[174]. Other possible mechanisms include improvements in insulin sensitivity, immune function/surveillance, and antioxidant defense capacity as well as alterations in gene function or apoptosis [175],[176]. Studies have also highlighted a potential role for epigenetic mechanisms which could reduce breast cancer risk in physically active women, including an increase in LINE-1 (long interspersed nucleotide elements-1) methylation (index of global DNA methylation) and an increase in the methylation of tumor-suppressor genes [176],[177]. Moderate levels of PA may also increase the expression of telomere-stabilizing proteins, thereby attenuating the effects of aging on telomere length and potentially reducing the risk of age-related diseases such as breast cancer [178],[179].

PA could also influence breast cancer risk through its effect on weight loss and reduced levels of body fat. This means that distinguishing the independent effects of PA on breast cancer risk is difficult because body fat reduction impacts a range of putative breast cancer risk markers, including circulating levels of sex hormones, insulin-like growth factors, adipokines, and inflammatory mediators [173]. Elevated circulating levels of adipokines such as leptin, interleukin-6, and tumor necrosis factor-alpha and the acute phase protein C-reactive protein as well as reduced levels of adiponectin are associated with high levels of body fat [173],[180], whereas weight loss interventions involving PA evoke reductions in circulating levels of inflammatory markers and leptin while increasing circulating levels of adiponectin [181],[182]. Despite this, evidence from both human [173],[174] and animal [175],[183] studies suggests that regular aerobic exercise can induce changes in biological risk factors (for example, sex hormones, insulin sensitivity, antioxidant defense capacity, and intracellular signaling pathways) that are independent of PA-induced changes in body weight and body composition.

The studies outlined above indicate the interactions which occur between epithelial cells and between them and stromal cells such as macrophages, fibroblasts, and adipocytes (Figure 3). They indicate the potential for new approaches to prevention, although translation to the clinic will be difficult. An excellent discussion of the problems is given by Strasser-Weippl and Goss [184].

## Clinical application

### Guidelines

#### Preventive therapy

Several guidelines advise how we might apply the knowledge that we have gained concerning hormonal prevention (tamoxifen, raloxifene, exemestane, and anastrozole) and lifestyle factors (weight control, exercise, and limitation of alcohol) to populations of women. Hormonal chemoprevention is suggested for women at increased risk, whereas lifestyle factors can be applied to all women since all are at some risk of breast cancer, and even at low risk, lifestyle factors are similar to those which help prevent other conditions such as CVDs and diabetes.

Three major sets of clinical guidelines were published concerning the selection of women for chemoprevention in 2013. The US Preventive Service Task Force gives guidelines for prescription of medication for risk reduction of breast cancer [185]. The recommendation applies to asymptomatic women 35 years or older without a prior diagnosis of breast cancer, ductal carcinoma *in situ*, or lobular carcinoma *in situ*. They advise use of the Gail model to assess risks and a cutoff of 1.66% 5-year risk. However, taking toxicity into account, they suggest that a threshold for advising treatment of 3% 5-year risk may be more appropriate and advise use of the tables published by Freedman and colleagues [186] and, as in the tables, that the balance for use/no use depends on age, race/ethnicity, the medication used, and whether the woman has a uterus.

The American Society of Clinical Oncology published their clinical practice guideline in August 2013 [187]. The report included a systematic review of randomized controlled trials and meta-analyses published between 2007 and 2013 which identified 19 trials and six chemoprevention agents. In women who are at increased risk of breast cancer and who are more than 35 years old, they suggest that tamoxifen (20 mg per day for 5 years) be discussed as an option to reduce the risk of ER^+^ breast cancer. In post-menopausal women, raloxifene (60 mg per day for 5 years) and exemestane (25 mg per day for 5 years) should also be discussed as options for breast cancer risk reduction. Those at increased breast cancer risk are defined as individuals with a 5-year projected absolute risk of breast cancer of more than 1.66% (based on the National Cancer Institute Breast Cancer Risk Assessment Tool or an equivalent measure) or women diagnosed with lobular carcinoma *in situ*. SERMs are not recommended for use in women with a history of deep vein thrombosis, pulmonary embolus, stroke, or transient ischemic attack or during prolonged immobilization or in combination with HRT. In this update of the guideline published in 2009, the phrase ‘may be offered’ was replaced by ‘should be discussed as an option’ in women at increased risk of breast cancer [187]. The American Society of Clinical Oncology reviewers concluded that ‘research is needed to address the many unresolved issues related to the poor uptake of breast cancer chemoprevention agents in women who are at increased risk. These include (1) the design of effective tools and approaches to educate providers on the option of chemoprevention, (2) efficacious interventions that communicate to eligible women the risks and benefits of specific chemoprevention agents, (3) the development of tools that more accurately identify women at increased risk, and (4) a greater understanding of what disparities and barriers exist with regard to chemoprevention use among women at higher risk for breast cancer’ [187]. The document provides in-depth reviews of all of the important trials.

The UK National Institute of Health and Care Excellence published guidelines for women at increased risk of breast cancer by virtue of a family history of the disease [188]. For the first time in the UK, their recommendation was that women at greater than 30% (1 in 3-4+) lifetime risk of breast cancer be ‘offered’ tamoxifen or raloxifene and that in those at greater than 17% (1 in 6+) lifetime risk preventive therapy be ‘considered’ for treatment. They did not endorse use of AIs, since the IBIS-2 study had not been published at the time, but did suggest that a lifestyle advice leaflet be given.

#### Lifestyle change

The ACS published guidelines on nutrition and PA measures for cancer prevention in 2012 [189]. The guidelines were based on published data. Randomized controlled trials were given greatest credence and cohort studies over case-control studies. Four lifestyle choices were recommended to reduce cancer risk: (a) achieve and maintain a healthy weight throughout life, (b) adopt a physically active lifestyle, (c) consume a healthy diet, with an emphasis on plant foods, and (d) limit consumption of alcoholic beverages.

Importantly, recommendations were also made for introduction of the guidelines into the community: ‘Public, private, and community organizations should work collaboratively at national, state, and local levels to implement policy and environmental changes…’ [190].

The Second WCRF/AICR Expert Report (Food, Nutrition, Physical Activity, and the Prevention of Cancer: A Global Perspective) was published in 2007 [117] and is continually updated [190]. The recommendations are similar to the ACS guidelines and are relevant to the prevention of other conditions such as CVD.

### Implementation

The guidelines outlined above are based on the best available knowledge and seem eminently sensible. It is widely appreciated that their implementation is a major and long-term problem. Although several models give reasonable indicators of risk of breast cancer, detecting women at risk in the population is problematic. For example, women with only a minor family history but with endocrine risk factors are very often not aware of their breast cancer risks. One solution is to use mammographic screening programs as a time to communicate risk information (including lifestyle parameters) and to highlight/signpost access to preventive therapy lifestyle programs [58],[191]. In a program in Manchester, UK, collecting risk information at screening was shown to be feasible, and 95% of women indicated that they wished to know their risks of breast cancer [58]. Women at high risk can be offered preventive therapy in the context of specialist clinics, but on a population basis it may be optimal to implement risk assessment and treatment in general practitioner practices as is the case for the prevention of CVD in clinical practice.

**Figure 4:**
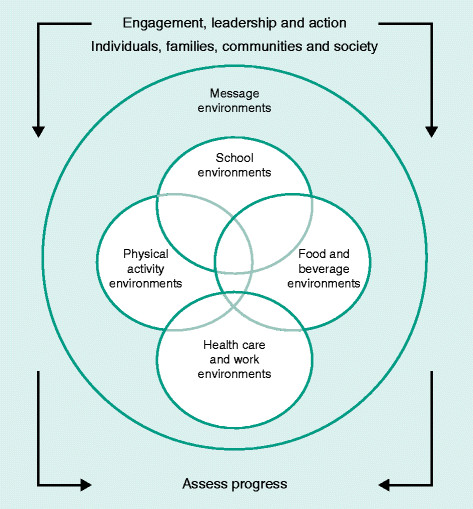
**US Institute of Medicine blueprint for lifestyle change.** Reprinted with permission from the US Institute of Medicine [192].

For lifestyle change, the goals for breast cancer prevention are the same as those required to solve the obesity epidemic. These are very well highlighted in the goals set by the US Institute of Medicine report on ‘Accelerating Progress in Obesity Prevention: Solving the Weight of the Nation’ [192]. The goals of the program encompassed integrating PA as a routine into everyday life, making healthy foods and beverages available everywhere, marketing messages pertaining to healthy nutrition and PA and expansion of the role of health-care providers, insurers, employers, and schools as national focal points for obesity prevention. The national (US) progress of this very broad and crucial program was summarized in a recent workshop [193]. The US Institute of Medicine believes that the obesity problem will be solved only by mobilizing the population of all ages for there to be an accelerated transformation to the obesity problem (Figure 4). The documents suggest groundbreaking approaches; similar ones could be adapted to other developed and developing countries.

Colditz and colleagues [194] recently summarized the critical barriers to change for the prevention of cancer in general. These included (a) skepticism that cancer can be prevented, (b) the short-term focus of cancer research, (c) interventions deployed too late in life, (d) research focus on treatment not prevention, (e) debates among scientists, (f) societal factors which affect health outcomes, (g) lack of transdiciplinary approaches, and (h) the complexity of successful implementation. These are barriers to be overcome.

## Conclusions

One conclusion of this review is that the application of measures that are already available, such as chemoprevention and lifestyle prevention, would result in appreciable reductions in breast cancer risk. A second conclusion is that the pace of advance of our understanding of the biology of breast cancer risk and development is highly likely to give rise to new avenues for prevention over the next 10 years. A major problem is applying what we already know concerning the efficacy of prevention to appropriate populations of women. To apply chemoprevention, we need to have measures in place to assess risk and to explain the pros and cons of treatment and for prescription of appropriate therapies. Lifestyle change is a population problem which involves publicity concerning its risks and benefits of change and providing mechanisms to support women in their choices throughout society as highlighted in the US Institute of Medicine documents.

## Authors’ original submitted files for images

Below are the links to the authors’ original submitted files for images. Authors’ original file for figure 1 Authors’ original file for figure 2 Authors’ original file for figure 3 Authors’ original file for figure 4

## Abbreviations

ACS: American cancer society
AI: Aromatase inhibitor
AICR: American institute for cancer research
AUC: Area under the receiver operating curve
BI-RADS: Breast imaging reporting and data system
BMI: Body mass index
BOADICEA: The breast and ovarian analysis of disease incidence and carrier estimation algorithm
*BRCA1/2*: Breast cancer 1/2
CI: Confidence interval
C-statistic: Area under the receiver operating curve
CVD: Cardiovascular disease
ER: Estrogen receptor
HRT: Hormone replacement therapy
IBIS: International breast cancer intervention study
IGF-1: Insulin-like growth factor-1
JNK: c-Jun N-terminal kinases
mTOR: mammalian target of rapamycin
PA: Physical activity
PR: Progesterone receptor
SERM: Selective estrogen receptor modulator
SNP: Single-nucleotide polymorphism
STAR: Study of tamoxifen and raloxifene
WCRF: World cancer research fund
WHI: Women’s health initiative
