# Supplementary figures and images for: Risk determination and prevention of breast cancer
Source: Breast Cancer Res. 2014 Sep 28;16:446. doi: 10.1186/s13058-014-0446-2 (PMC4303126; doi:10.1186/s13058-014-0446-2)

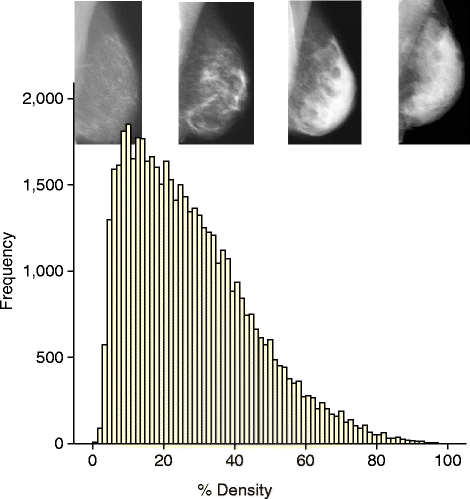

Supplement: Supplementary file 1 — Authors’ original file for figure 1 [file 13058_2014_446_MOESM1_ESM.gif]

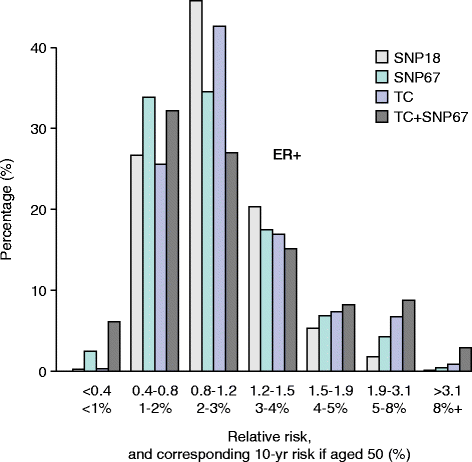

Supplement: Supplementary file 2 — Authors’ original file for figure 2 [file 13058_2014_446_MOESM2_ESM.gif]

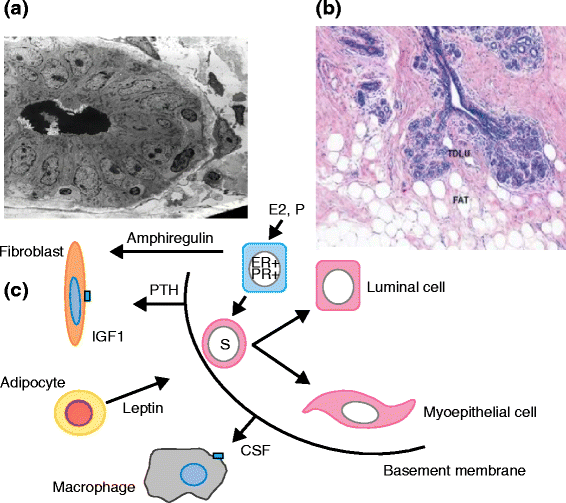

Supplement: Supplementary file 3 — Authors’ original file for figure 3 [file 13058_2014_446_MOESM3_ESM.gif]

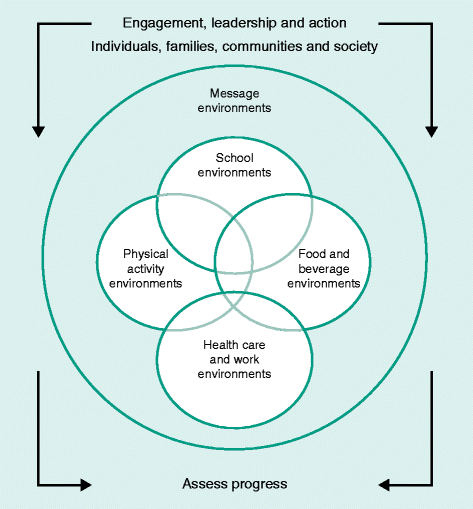

Supplement: Supplementary file 4 — Authors’ original file for figure 4 [file 13058_2014_446_MOESM4_ESM.gif]
